# High Prevalence of Strongyloidiasis in Spain: A Hospital-Based Study

**DOI:** 10.3390/pathogens9020107

**Published:** 2020-02-11

**Authors:** Ana Requena-Méndez, Joaquin Salas-Coronas, Fernando Salvador, Joan Gomez-Junyent, Judith Villar-Garcia, Miguel Santin, Carme Muñoz, Ana González-Cordón, Maria Teresa Cabezas Fernández, Elena Sulleiro, Maria del Mar Arenas, Dolors Somoza, Jose Vazquez-Villegas, Begoña Treviño, Esperanza Rodríguez, Maria Eugenia Valls, Jaume Llaberia-Marcual, Carme Subirá, Jose Muñoz

**Affiliations:** 1 Barcelona Institute for Global Health, ISGlobal-Hospital Clinic, Universitat de Barcelona, 08036 Barcelona, Spain; carme.subira@isglobal.org (C.S.); jose.munoz@isglobal.org (J.M.); 2 Department of Global Public Health, Karolinska Institutet, 171 77 Solna, Sweden; 3 Tropical Medicine Unit. Hospital de Poniente, El Ejido, 04700 Almería, Spain; joaquinsalascoronas@yahoo.es (J.S.-C.); tcabezasf@yahoo.es (M.T.C.F.); 4 Department of Infectious Diseases, Vall d’Hebron University Hospital, PROSICS Barcelona, 08035 Barcelona, Spain; fmsalvador@vhebron.net; 5 Department of Infectious Diseases, Hospital Universitari Bellvitge, 08907 Barcelona, Spain; gjunyent@hotmail.com (J.G.-J.), msantin@bellvitgehospital.cat (M.S.); 6 Infectious Diseases Department, Hospital del Mar-IMIM, 08003 Barcelona, Spain; JVillar@parcdesalutmar.cat (J.V.-G.); marenas@parcdesalutmar.cat (M.d.M.A.); 7 Department of Microbiology, Hospital Sant Pau, 08041 Barcelona, Spain; CMunoz@santpau.cat (C.M.); JLlaberia@santpau.cat (J.L.-M.); 8 Department of Infectious Diseases, Hospital Clinic, 08036 Barcelona, Spain; AGONZAL1@clinic.cat; 9 Department of Microbiology, Vall d’Hebron University Hospital, PROSICS Barcelona, 08035 Barcelona, Spain; esulleiro@vhebron.net; 10 Department of Microbiology. Hospital Universitari Bellvitge, 08907 Barcelona, Spain; dsomoza@bellvitgehospital.cat; 11 Tropical Medicine Unit, Distrito Poniente, 04700 Almería, Spain; pepevazquez@ya.com; 12 Tropical Medicine Unit Vall d’Hebron-Drassanes, PROSICS Barcelona, 08035 Barcelona, Spain; btrevino.bcn.ics@gencat.cat; 13 Parasitology Department, Centro Nacional de Microbiologia—Instituto de Salud Carlos III, 28020 Madrid, Spain; erodrgez@isciii.es; 14 Department of Microbiology, Hospital Clínic, Barcelona 08036, Spain; mevalls@clinic.cat

**Keywords:** strongyloidiasis, *Strongyloides stercoralis*, prevalence, migrants, Spain

## Abstract

*Introduction*: Strongyloidiasis is a prevailing helminth infection ubiquitous in tropical and subtropical areas, however, seroprevalence data are scarce in migrant populations, particularly for those coming for Asia. *Methods*: This study aims at evaluating the prevalence of *S. stercoralis* at the hospital level in migrant populations or long term travellers being attended in out-patient and in-patient units as part of a systematic screening implemented in six Spanish hospitals. A cross-sectional study was conducted and systematic screening for *S. stercoralis* infection using serological tests was offered to all eligible participants. *Results*: The overall seroprevalence of *S. stercoralis* was 9.04% (95%CI 7.76–10.31). The seroprevalence of people with a risk of infection acquired in Africa and Latin America was 9.35% (95%CI 7.01–11.69), 9.22% (7.5–10.93), respectively. The number of individuals coming from Asian countries was significantly smaller and the overall prevalence in these countries was 2.9% (95%CI −0.3–6.2). The seroprevalence in units attending potentially immunosuppressed patients was significantly lower (5.64%) compared with other units of the hospital (10.20%) or Tropical diseases units (13.33%) (*p* < 0.001). *Conclusions*: We report a hospital-based strongyloidiasis seroprevalence of almost 10% in a mobile population coming from endemic areas suggesting the need of implementing strongyloidiasis screening in hospitalized patients coming from endemic areas, particularly if they are at risk of immunosuppression.

## 1. Introduction

Migration flows from Latin American, African and Asian countries to Europe have shown that a high percentage of arriving individuals may be chronically infected with *Strongyloides stercoralis*, which may have a public health impact in non-endemic countries that are hosting these populations [1,2]. The infection is ubiquitous in tropical and subtropical areas, although it may also occur in temperate countries with appropriate conditions such as certain areas of Spain or Italy [3,4,5]. Worldwide estimates based on standard fecal techniques have suggested that between 30 and 100 million people are infected worldwide. These figures may be underestimates due to the low sensitivity of traditional diagnostic methods [1,6].

Unlike other parasitic infections, this helminth has some characteristics that are of particular importance for migrant populations [7]. Firstly, the infection can persist for the whole lifetime due to the possibility of causing autoinfection in the human host [8]. Through this phenomenon, the filariform larvae penetrate intestinal mucosa in the large intestine or perianal skin and migrate to complete another lifecycle. Therefore, people coming from endemic areas may be at risk for their whole life, irrespective of the moment they arrive in a non-endemic area, as long as they are not treated. Secondly, *Strongyloides stercoralis* infection is generally asymptomatic or causes unspecific symptoms, and thus goes unnoticed by health professionals who are not looking for it [9]. Thirdly, although the infection is rarely transmitted from person to person [10], it can be transmitted through organ transplantation, and autochthonous cases have been reported in non-endemic areas [11]. Therefore, screening should be considered for potential donors at risk of the infection [12,13,14]. Finally, in the case of immunosuppression, particularly those displaying a concomitant use of steroids, transplant recipients, or patients with malignancies and Human T-Cell Lymphotropic virus-1 co-infections, the parasite may enter into a high replicating cycle (called hyperinfection) or disseminate to vital organs (disseminated strongyloidiasis), causing a severe disease with a high mortality [15].

The diagnosis of strongyloidiasis in non-endemic areas is currently based on a serological test, which has a considerably higher sensitivity compared with standard fecal techniques [8]. Despite having cross-reactions with other helminthic infections, this is less likely to occur in migrant populations since the possibility of co-infections is lower [16] and it is thus now the current recommended screening technique for these populations [17]. The sensitivity of the serological tests in immunosuppressed individuals seems to be lower [18], but only limited data are available and further prospective studies should better evaluate the accuracy of serological tests in immunosuppressed patients. 

Even with the limitations of current diagnostic methods, the screening of high-risk groups and treatment of infected individuals are of key importance. In this regard, the screening of strongyloidiasis in newly arrived migrants has been recommended by the European Centre for Disease Prevention and Control [19,20], particularly in immunosuppressed individuals, given the potential individual morbidity and mortality [16].

Evidence of the seroprevalence of strongyloidiasis in migrant populations is scarce, particularly if it is assessed in Asian countries. Available data suggest that it is known to vary substantially, depending on the country of origin, being particularly high in people coming from countries such as Cambodia (36%) or Latin American countries (26%) [21]. Hospital-based prevalence studies conducted in specialized units have suggested that the prevalence of *S.stercoralis* is between 4.5% and 11% in migrant populations [22,23,24]. Available data suggest a higher prevalence of infection in immunosuppressed individuals at risk [25,26,27]. 

Our study aims to evaluate the prevalence of *S. stercoralis* at the hospital level in migrant populations or long-term travelers being attended to in out-patient and in-patient units as part of the systematic screening implemented in six Spanish hospitals.

## 2. Results

From October 2014 to September 2016, 2024 individuals attended to in the six hospitals were screened for strongyloidiasis and 77 of them were excluded from the study because either the screening had not been done with a serological test or it could not be assured that a systematic approach had been used in several units of one hospital. Therefore, 1948 individuals were finally included. Basic demographic data are summarized in Table 1.

The median age was 38 years (Interquartile range 31–47) and 57.96% were male; 804 (41.27%) were screened at infectious disease or tropical disease units, 709 (36.40%) in HIV-units, 150 (7.70%) in transplant units, 101 (5.18%) in oncology or hematology units, and 86 (4.41%) in rheumatology or autoimmune disease departments or other units specifically addressing potentially immunosuppressed patients. Finally, 77 (3.95%) and 21 (1.08%) were recruited in general services of the hospital (e.g., emergency and internal medicine) and in 21 (1.08%), the department of origin was unknown. Of the total screened population, 58.96% (1106/1876) had a Latin-American origin, 31.98% (600/1876) were from Africa, 5.49 (103) were from Asia and 3.57% (67) were from Europe.

There were differences across the centers concerning the country of origin of individuals screened. This could be explained by the demographics of the migration flows in Spain: In the hospital located in Almeria, which is very close to the African border, 94.39% had an African origin, whereas for others hospitals, more than half of individuals had a Latin-American origin (Table 1). Services/units of hospitals were also grouped into those units attending to essentially immunosuppressed or potentially immunosuppressed patients (HIV, transplant, haematology, oncology, and autoimmune disease units) compared with units that were not attending to immunosuppressed patients (infectious disease units, tropical disease units, or general services of the hospital). Latin American migrants were slightly more frequently included in the potentially immunosuppressed group (67.19%) compared with the other group (49.12%) (*p*-value < 0.001).

### 2.1. Seroprevalence 

Of the 1947 patients tested, 176 had a positive serological test for *S. stercoralis*. The overall seroprevalence of *S. stercoralis* was 9.04% (95%CI 7.76–10.31). The prevalence varied significantly among the centers, from 6.57% (13/198) in Hospital del Mar to 29.69% (19/64) in Hospital de Bellvitge (*p* < 0.001). Women had a higher seroprevalence (10.77%) compared with men (6.96%) (*p*-value = 0.005). We found no association between the age group and the prevalence (*p* = 0.962). There were 33/176 (18.75%) individuals with *Strongyloides* that were considered immunosuppressed. There were three transplant patients, three patients with neoplasia, 10 patients with an autoimmune disease, two patients under treatment with steroids for other reasons, and 14 patients with HIV and with <500 CD4 (one of whom had <200 CD4). There were an additional 17 patients with HIV and >500 CD4 that were not considered immunosuppressed individuals. No patients developed hyperinfection or disseminated disease.

#### 2.1.1. Seroprevalence by Geographic Distribution

The seroprevalence of people with a risk of infection acquired in Africa was 9.35 (95%CI 7.01–11.69), with Sub-Saharan Africa (SSA) countries exhibiting a higher value (10.89%; 95%CI 8.03–12.75) than North African countries (4.29%; 95%CI 0.8–7.69) (*p* = 0.019).

**Figure 1:**
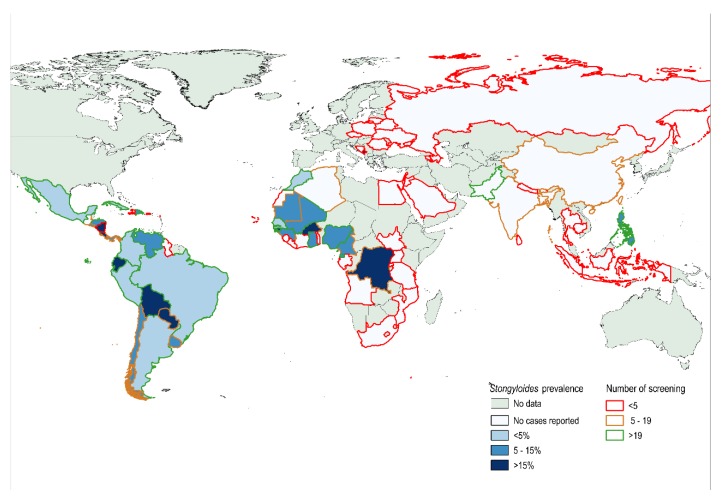
Identified seroprevalence of strongyloidiasis in migrants and travelers in Spain by country of acquisition (The figure was built with the QGIS software, version 2.18.22).

Morocco was the country of acquisition from Africa with the largest population screened, showing a seroprevalence of 4.69% (95%CI 0.9–8.34). There were only eight countries with ≥20 observations (See Figure 1) and the results of prevalence by country of origin are summarized in the Appendix (Table A1).

The Latin America (LA) continent showed a seroprevalence of 9.22% (95% CI 7.5–10.93), with the seroprevalence in people coming from South American countries being 9.64% (95% CI 7.74–11.5) and the prevalence in people coming from Central America and the Caribbean being 6.98% (95% CI 3.13–10.82) (*p* = 0.268). Ecuador and Bolivia were the countries of acquisition with the highest seroprevalence, with values of 17.48% (95% CI 10.01–24.9) and 15.81% (95% CI 11.84–19.77), respectively. The prevalence was below 8% for the other countries, with more than 50 people being screened. (see Figure 1)

The number of individuals screened coming from Asian countries was significantly smaller and the overall prevalence in these countries was 2.9% (95%CI−0.3, 6.2). Only three cases were observed in people coming from South-East Asia (11.11%; 95%CI 1.5–23.78) and all three were originally from the Philippines (seroprevalence 13.63%).

There was only one case (1/14 (7.14%) from an individual from East European countries. The patient was from Montenegro and he had not travelled outside Europe. 

There were 50 individuals originally from West Europe that were also screened for *S*.*stercoralis* after long-term travels to endemic countries. In the three out of 50 individuals (5.6%) with a positive result, the country of acquisition could not be identified since all of them had undertaken trips to several endemic areas in the past (Latin America and South East Asia countries).

#### 2.1.2. Seroprevalence by Screening Department

The seroprevalence in units attending to immunosuppressed and potentially immunosuppressed patients was significantly lower (5.64%) compared with the seroprevalence in other units of the hospital (10.20%) or tropical disease units (13.33%) (*p* < 0.001) (See Table 2). HIV and transplant units showed the lowest prevalence rates (4.51% and 2%, respectively).

In these units, the rate in individuals with an LA origin was 3.74% and 3.39%, respectively, which are significantly lower than the results of other units.

## 3. Discussion

Our study shows an overall prevalence of almost 10%, which is consistent with other studies conducted among migrant population living in non-endemic areas [17].

This is one of the first studies to evaluate the results of a systematic screening in potentially immunosuppressed patients in which the disease may be more severe.

The high prevalence found in our study supports the need for screening strategies in patients that are potentially immunosuppressed particularly for Sub-Saharan African and Latin American migrants.

Potentially immunosuppressed people are at a higher risk of developing a severe complication of the disease, as has been extensively reported elsewhere [15]. However, the probability of developing hyperinfection or dissemination of the infection in the high-risk immunosuppressed population is unclear. Systematic screening has recently been recommended in high-risk population coming from endemic areas [19]. However, few programs have reported their results so far. In this regard, a reference center in Austria has initiated the screening of *S. stercoralis* for all transplant recipients showing a 3% prevalence even though only a small percentage are migrants and most of them are from East European countries [28].

Interestingly, the seroprevalence in Ecuador and Bolivia was much higher compared with other LA countries, but similar compared with other studies conducted in migrants from these countries [29], which may be partially explained by the profile of migrants coming from rural areas in these countries. In addition, the sampling in these countries may also have been overrepresented compared to other LA countries and the prevalence in LA may thus have been overestimated.

In South-East Asia and North Africa, our data show a considerable seroprevalence rate, being higher than 5% in countries such as Morocco or the Philippines. However, the small number of individuals screened coming from these countries is very low, which may prevent proper conclusions from being drawn regarding the seroprevalence in these geographic areas. Published data on the *Strongyloides* prevalence in Asian countries are very scarce and inexistent in other countries (Morocco and the Philippines). Although we have not found studies specifically providing prevalence estimates in Asian migrants, some surveillance data about health conditions in migrants have reported a 5%–7% rate of strongyloidiasis among people coming from South-East Asia and South Asian countries [30]. Further studies conducted in non-endemic areas should better evaluate the *S.stercoralis* prevalence in people coming from Asian countries.

The seroprevalence in women was found to be significantly higher than that of men, which differs from other studies, where the prevalence was more frequent in males [31]. One possible explanation for this is the large contribution of Bolivian women to the population tested in our dataset, which have a high seroprevalence. 

In our study, the prevalence in HIV-infected individuals and patient from transplant units, but not necessarily immunosuppressed individuals, was much lower compared with other general out /in-patient unit services of the hospitals. One possible explanation that could have contributed to the lower prevalence found in potentially immunosuppressed patients is that the serology had a lower sensitivity in immunosuppressed patients, as has been reported in other studies [18]. The sensitivity of the serology in immunosuppressed patients deserves a further evaluation to examine its accuracy, since only a few studies have evaluated it, showing a lower sensitivity compared to parasitological techniques [18], and no studies have evaluated, its accuracy at different levels of immunosuppression. In addition, the accuracy of the serological test in the diagnosis of strongyloidiasis in immunosuppressed patients, and which is the best strategy for screening in such cases, is still unclear. [16].

Unfortunately, and due to the study design, we could not obtain any information about the level of immunosuppression that patients had at the time the screening test was performed. Therefore, we could not estimate to what extent the lower-sensitivity of the screening test causes this difference. Further prospective studies should better evaluate the accuracy of the serological test in immunosuppressed patients, including an assessment of the accuracy with different levels or categories of immunosuppression.

It must be added that the prevalence of Latin-American migrants screened in these units (HIV and transplant) was particularly low compared with other units of screening. The different profile of Latin American migrants attended to in the HIV and transplant units compared with tropical units (e.g., migrant origin from urban vs. rural areas) could partially explain this. 

This is a hospital-based study and the results should be interpreted in the context of a hospital-based population. Generalizing our prevalence results to the general (or wider) population should be done with caution since the positive selection bias should be taken into account. In addition, another limitation is the difference in the prevalence between the hospitals participating in this study that could be partially explained by differences in the migrant profile. However, a selection bias may have been introduced if the screening was not systematic in all at-risk-individuals, which could have overestimated the prevalence in some units.

## 4. Materials and Methods 

### 4.1. Study Population, Data Collection and Patient Management

This is a cross-sectional study, carried out as part of a hospital-based prospective cohort study conducted in six referent hospitals in Spain (Hospital Clinic, Hospital del Mar, Hospital Universitari Bellvitge, Hospital Sant Pau and Hospital Vall d’Hebron located in Barcelona and Hospital de Poniente in Almeria province) aimed to evaluate the prevalence and risk factors associated with *Strongyloides stercoralis* infection, particularly those related to immunosuppressed patients.

Although systematic screening has not been widely implemented at a national level, it was established that individuals either hospitalized or being attended to in any specialized out-patient or in-patient units of the hospital should be screened for strongyloidiasis. In particular, infectious diseases or tropical disease units attending to potentially severe immunosuppressed patients (Hematology, Oncology, autoimmune diseases/Rheumatology, and HIV and transplant units) were willing to participate in and implement the systematic screening. In these units, the screening was conducted irrespective of whether the individual was immunosuppressed or not. However, it should be added that the reasons for hospitalization in those units were always conditions related to such units. Not all hospital units or departments participated in the study.

For that purpose, training sessions were organized in those units attending to potentially immunosuppressed patients to enhance the importance of *Strongyloides* screening and the physicians in charge of the patient ordered the serological test. 

Migrant adult individuals or adult long-term travelers (defined as those staying one year or more in any endemic country) were systematically offered screening for *S. stercoralis* based on a serological test. Endemic countries were considered any country in Asia, Oceania, Africa, East Europe, or Latin America countries. Therefore, inclusion criteria were having resided in an endemic country for one more than year and having been attended to in the hospital for any reason, irrespective of the level of immunosuppression.

As part of this program, all individuals who met the inclusion criteria for screening were offered a serological blood test for *S. stercoralis* infection. All positive patients were invited to participate, signed the consent form, and were provided treatment with ivermectin 200 mcg/kg over two consecutive days. Information on aspects related to immunosuppression and other clinical and laboratory aspects were collected in a questionnaire. Participants were clinically and microbiologically followed-up with a serological test and with three stool tests if the initial stool test was positive after six months of therapy as part of the routine clinical practice. 

### 4.2. Microbiological Procedures Serology of S. stercoralis

Serum samples were tested for specific antibodies using a commercial Enzyme Linked Immunosorbent assay (ELISA) according to the manufacturer’s instructions. A single ELISA test was used (Strongyloidiasis ELISA Kit based on IVD *Strongyloides stercoralis* crude antigen, SCIMEDX, Dover, NJ, USA). To reduce the inter-laboratory variability, an internal workshop was carried out before starting the study to standardize the serological techniques and the interpretation of the results. Positive samples were defined by absorbance greater than 0.2 OD units. The absorbance of the study sample/0.2 ≥1 (calculated value) was used as the cut-off. The sensitivity and specificity for this test using this cut-off have been reported to be 91.2% and 99.1% respectively [32].

### 4.3. Sample Size Estimation

Based on other seroprevalence studies conducted in non-endemic areas, we estimated a prevalence of *S. stercoralis* of 11% for our cohort [17]. Based on these data, 1700 individuals were estimated to be screened during 24 months to recruit 150 cases of strongyloidiasis, considering an Alfa error of 5% and a lost to follow-up rate of 10%.

### 4.4. Statistical Analysis

Categorical variables were described using a frequency distribution and the median and interquartile range were used to describe age. Countries of origin were subsequently grouped into different geographic areas according to the criteria that could more precisely identify the migration patterns. The GeoSentinel geographic area distribution was selected [33]. Prevalence point estimates and their 95% confidence intervals (95%CI) were obtained. We used Fisher’s exact test to compare percentages. Data were managed and analyzed using STATA 13 (StataCorp LLC, Lakeaway, Texas USA). We built a world map with country of acquisition’s prevalence using the free software QGIS, version 2.18.22. Hospital units identified as attending to potentially immunosuppressed patients were: hematology, oncology, HIV, autoimmune diseases, rheumatology, transplant units and oncology.

### 4.5. Ethics

This project was part of a study where individuals with a positive for *S. stercoralis* test were further invited to participate in a study to evaluate risk factors associated with infections particularly related to immunosuppression and signed a written consent form. The study was approved by the ethic committee of all hospitals participating in the study (Hospital Clinic HCB/2014/0321). Only adult individuals participated in this study. The study has been reported following the Strengthening the Reporting of Observational Studies in Epidemiology (STROBE) statement for reporting observational studies (Table S1).

## 5. Conclusions

In this paper, we reported a hospital-based systematic screening of *Strongyloides* with a seroprevalence of almost 10% in the migrant population from endemic areas, and thus presented evidence for the need to implement strongyloidiasis screening strategies in hospitalized patients. Even though such a high rate was not found in the potentially immunosuppressed group of people, screening should be particularly considered in these cases due to the clinical impact of hyperinfection or disseminated infection in this population, and also because of a lack of accurate data on serological testing in immunosuppressed people.

## Figures and Tables

**Table 1:** General characteristics of individuals screened for strongyloidiasis.

Variable	Frequency
**Age (n = 1943)**	
Median (IQR)	38 (31–48)
Group	
<25	201 (10.34%)
26–39	846 (43.54%)
40–54	652 (33.56%)
>54	244 (12.56%)
**Male sex (n = 1634)**	**947 (57.96%)**
**Hospitals**	
Clinic	902
Vall d’Hebron	411
Hospital Poniente	303
Hospital Mar	198
Hospital Sant Pau	70
Hospital Bellvitge	64
**Departments or units**	
General services	77
Autoimmune/Rheumatology	86
Transplant units	150
Hemato-Oncology	101
International Health	804
HIV units	709
Other	21
**Continents (n = 1876)**	
Africa	600
North Africa	141
SSA	459
America	1106
South America	934
Central America &Caribe	172
Asia	103
South-Central Asia	61
South-East Asia Middle East	273
East Asia	12
Europe	67
West Europe	53
East Europe	14

**Table 2:** Seroprevalence by geographic area (GeoSentinel areas).

	Percentage (CI)	A: Immunosuppressed Units * (%)	B: Other Non Immunosuppressed Units from the Hospital ** (%)	C: Tropical Diseases Units	*p*-Value ***
South-America	9.6 (7.8–11.7)	30/558 (5.38%)	1/11 (9.1)	59/365 (16.2%)	<0.001
Central America and Caribbean	7 (3.7–11.9)	5/128 (3.91)	0/8 (0)	7/36 (19.44)	0.04
North African	4.3 (1.6–9.1)	3/90 (3.3)	0/3	3/47 (6.4)	0.658
Sub-Saharan Africa	10.9 (8.2–14.1)	12/155 (7.7)	1/6 (16.7)	37/298 (12.4)	0.286
Middle East	0 (0–70.6)	0/2	0	0	---
South-Central Asia	0 (0–58.7)	0/29	0/5	0/27	
South-East Asia	11.1 (2.4–29.2)	2/19 (10.5)	0/2	1/6 (16.7)	0.801
East Asia	0 (0–26.4)	0/5	0	0/7	---
East Europe	7.1 (0.2–33.9)	1/13 (7.7)	0/1	0	0.773
Western Europe (travellers with multiple travels)	5.7 (1.2–15.7)	3/21 (14.3)	0/15	0/17	0.089
Total	9 (7.7–10.3)	59/1046 (5.64)	10/98 (10.2)	107/803 (13.39	<0.001

* Immunosuppressed units: Haematology, Oncology, Autoimmune diseases, Rheumatology, HIV and Transplant Units; ** Other non-immunosuppressed units: Internal Medicine, Emergency, Cardiology, Neurology, Dermatology and Pneumology; *** Chi square test used to calculate difference in prevalence in units A, B and C.

## Supplementary Materials

The following are available online at https://www.mdpi.com/2076-0817/9/2/107/s1, Table S1: STROBE statement—Checklist of items that should be included in reports of cross-sectional studies.

## Appendix

**Table A1:** Seroprevalence of *S. stercoralis* infection by country of origin.

	Prevalence	CI-95%	n
**LATIN AMERICA**			
ARGENTINA	3.4	0.7–9.5	89
BOLIVIA	15.8	12–20.2	329
BRAZIL	2.73	0.6–7.8	110
COLOMBIA	3.8	1–9.5	105
CHILE	5.6	0.1–27.3	18
CUBA	2.6	0.07–13.5	39
ECUADOR	17.5	10.7–26.2	103
MEXICO	4.5	0.1–22.8	22
PARAGUAY	18.2	2.3–51.8	11
PERU	1.3	0.03-0.7	75
VENEZUELA	5.1	1.4–12.6	78
**AFRICA**			
ANGOLA	0	0–70.6 *	3
ALGERIA	0	0–45.9 *	6
BURKINA FASO	16.7	2.1–48.4	12
CABO VERD	0	0–97.5 *	1
CAMEROON	14.3	1.8–42.8	14
CONGO	33.3	4.3–77.7	6
EGYPT	0	0–84.2 *	2
GAMBIA	8.7	1.1–28	23
IVORY COAST	0	0–52.2 *	5
EQUATORIAL GUINEA	13.6	7–23	81
GABON	0	0–84.2 *	2
GHANA	14.8	4.2–33.7	27
GUINEA BISSAU	25	13.2–40.3	44
GUINEA CONAKRY	12.5	15.5–38.3	16
LIBERIA	0	0–97.5 *	1
MAURITANIA	14.3	0.3–57.9	7
MALI	6.9	2.6–14.4	87
MORROCCO	4.7	1.7–9.9	128
MOZAMBIQUE	0	0–97.5 *	1
NIGERIA	5.6	0.7–18.7	36
RUANDA	0	0–84.2 *	2
SAUDI ARABIA	0	0–84.2 *	2
SENEGAL	4.9	1.34–12	82
TANZANIA	0	0–60.2	4
**ASIA**			
AZERBAIYAN	0	0–97.5 *	1
BANGLADESH	0	0–45.9 *	6
BUTAN	0	0–97.5 *	1
CAMBODIA	0	0–97.5 *	1
CHINA	0	0–26.5	12
INDONESIA	0	0–84.2 *	2
ISRAEL	0	0–97.5 *	1
JORDAN	0	0–97.5 *	1
INDIA	0	0–28.5 *	11
LEBANNON	0	0–97.5 *	1
NEPAL	0	0–84.2 *	2
PAKISTAN	0	0–9	39
PHILIPPINES	13.6	2.9–34.9	22
SRI LANKA	0	0–97.5 *	1
THAILAND	0	0–84.2 *	2
**EAST EUROPE**			
ARMENIA	0	0–97.5 *	1
BIELORRUSSIA	0	0–97.5 *	1
BOSNIA	0	0–97.5 *	1
LITHUANIA	0	0–97.5 *	1
MONTENEGRO	0	0–97.5 *	1
POLAND	0	0–97.5 *	1
CZECH REPUBLIC	0	0–97.5 *	1
ROMANIA	0	0–60.2	4
RUSSIA	0	0–97.5 *	1
UKRAINE	0	0–84.2 *	2

*: One-sided, 97.5% confidence interval.
