# Supplementary material for: High Prevalence of Strongyloidiasis in Spain: A Hospital-Based Study
Source: Pathogens. 2020 Feb 11;9(2):107. doi: 10.3390/pathogens9020107 (PMC7167856; doi:10.3390/pathogens9020107)
Supplement: Supplementary file 1 [file pathogens-09-00107-s001.pdf]

Supplementary file

Table S1. STROBE Statement—Checklist of items that should be included in reports of ***cross-sectional studies***

	Item No	Recommendation
**Title and abstract**	1	Indicate the study's design with a commonly used term in the title or the abstract: YES
		Provide in the abstract an informative and balanced summary of what was done and what was found: YES (section title)
**Introduction**		
Background/rationale	2	Explain the scientific background and rationale for the investigation being reported: YES (Section: Introduction, all paragraphs).
Objectives	3	State specific objectives, including any pre-specified hypotheses: YES (Section: Introduction, last paragraph)
**Methods**		
Study design	4	Present key elements of study design early in the paper: YES (Section: Study population, data collection and patient management, first paragraph)
Setting	5	Describe the setting, locations, and relevant dates, including periods of recruitment, exposure, follow-up, and data collection YES (Section: Study population, data collection and patient management, first paragraph)
Participants	6	Give the eligibility criteria, and the sources and methods of selection of participants. YES (Section: Study population, data collection and patient management, second and third paragraph)
Variables	7	Clearly define all outcomes, exposures, predictors, potential confounders, and effect modifiers. Give diagnostic criteria, if applicable. YES (Section: Statistical analysis, fourth paragraph)
Data sources/ measurement	8*	For each variable of interest, give sources of data and details of methods of assessment (measurement). Describe comparability of assessment methods if there is more than one group. YES (Section: Microbiological procedures serology of S. stercoralis, sixth paragraph and Statistical analysis, fourth paragraph)
Bias	9	Describe any efforts to address potential sources of bias
Study size	10	Explain how the study size was arrived at YES (Section: Sample size estimation, paragraph 2.)
Quantitative variables	11	Explain how quantitative variables were handled in the analyses. If applicable, describe which groupings were chosen and why YES (Section: statistical analysis, third and fourth paragraphs)
Statistical methods	12	(*a*) Describe all statistical methods, including those used to control for confounding YES (Section: statistical analysis, third and fourth paragraph)
		(*b*) Describe any methods used to examine subgroups and interactions YES (Section: Statistical analysis (fourth paragraph)
		(*c*) Explain how missing data were addressed
		(*d*) If applicable, describe analytical methods taking account of sampling strategy
		(*e*) Describe any sensitivity analyses
**Results**		
Participants	13*	(a) Report numbers of individuals at each stage of study—eg numbers potentially eligible, examined for eligibility, confirmed eligible, included in the study, completing follow-up, and analyzed YES (Section: Results, first paragraph)
		(b) Give reasons for non-participation at each stage (YES, Section: Results, first paragraph)
		(c) Consider use of a flow diagram
Descriptive data	14*	(a) Give characteristics of study participants (eg demographic, clinical, social) and information on exposures and potential confounders YES (Section: Results, first paragraph and table 1)
		(Indicate number of participants with missing data for each variable of interest (YES (Section: Results, first paragraph)
Outcome data	15*	Report numbers of outcome events or summary measures YES (Section: Results, second third and fourth paragraphs)
Main results	16	(*a*) Give unadjusted estimates and, if applicable, confounder-adjusted estimates and their precision (eg, 95% confidence interval). Make clear which confounders were adjusted for and why they were included YES (Section: Results, fourth and fifth paragraph)
		(*b*) Report category boundaries when continuous variables were categorized
		(*c*) If relevant, consider translating estimates of relative risk into absolute risk for a meaningful time period
Other analyses	17	Report other analyses done—eg analyses of subgroups and interactions, and sensitivity analyses YES (Section: Results, Seroprevalence by geographic distribution and Seroprevalence by screening department, all paragraphs)

**Discussion**		
Key results	18	Summarise key results with reference to study objectives. YES (Section: Discussion, first paragraph)
Limitations	19	Discuss limitations of the study, taking into account sources of potential bias or imprecision. Discuss both direction and magnitude of any potential bias YES (page 12)
Interpretation	20	Give a cautious overall interpretation of results considering objectives, limitations, multiplicity of analyses, results from similar studies, and other relevant evidence YES (Section Discussion, 10th paragraph)
Generalisability	21	Discuss the generalisability (external validity) of the study results YES (Section Discussion, 11th paragraph)
**Other information**		
Funding	22	Give the source of funding and the role of the funders for the present study and, if applicable, for the original study on which the present article is based YES (Sections: Acknowledgements and Financial disclosure form)
